# How Do Green-Leaf and Yellow Tea Cultivars Influence Black Tea Flavor? Insights from Metabolomic Analysis with Ninghong Tea-Processing Technologies

**DOI:** 10.3390/ijms27188313

**Published:** 2026-09-18

**Authors:** Xinfeng Jiang, Chen Li, Yanfang Yu, Lixian Wang, Xuezhen Yang, Xiaoling Wang

**Affiliations:** 1 Jiangxi Institute of Economic Crops, Nanchang 330202, China; hanwuji1110@126.com (C.L.); wanglixian0214@126.com (L.W.);; 2 Institute of Biological Resources, Jiangxi Academy of Sciences, Nanchang 333104, China

**Keywords:** Huangjinju, Ningzhou No. 2, widely targeted metabolomics, leaf color mutant, black tea, differential metabolites, flavor quality

## Abstract

To elucidate the metabolic mechanisms underlying black tea flavor formation driven by cultivar genotype and processing technology, two cultivars, the green-leaf “Ningzhou No. 2” and the yellow-leaf “Huangjinju”, were processed via the traditional Ningzhou Gongfu black tea manufacturing protocol. Ultra-performance liquid chromatography–tandem mass spectrometry (UPLC–MS/MS)-based widely targeted metabolomics was employed to compare the metabolites and main biochemical components in fresh leaves, combined with the sensory evaluation of processed black tea from *Camellia sinensis* cv. “Huangjinju” and “Ningzhou No. 2”. The results showed that a total of 2208 metabolites were detected, representing 25 classes, including flavonoids, phenolic acids, terpenoids, esters, amino acids and their derivatives, and alkaloids. The characteristic differential metabolites in the fresh leaves from the two cultivars were primarily flavonoids. After processing, “Huangjinju” exhibited a relatively higher content of terpenoids, whereas “Ningzhou No. 2” showed relatively higher levels of flavonoids and phenolic acids. “Huangjinju” excelled with the Ningzhou Gongfu method, emphasizing floral notes and a liquor color, a sweet and mellow taste, and spent leaves, yielding a richer sweet–floral profile with nutty undertones and a reddish liquor. Cultivar primarily influenced bitterness, while processing modulated floral, fruity, nutty, sweet, and off-flavors. Metabolic enrichment analysis revealed cultivar-specific differences in fatty acids, terpenoids, heterocycles, and flavonoids, aligning with sensory profiles. These findings provide a metabolic basis for optimizing Ningzhou Gongfu black tea processing and quality control. KEGG pathway enrichment analysis further identified that galactose metabolism was a key pathway underlying the metabolic differences between the black teas of the two cultivars. Metabolic differences between the two tea cultivars determined the quality and processing suitability of fresh leaves. During the processing of black tea, chlorophyll content is significantly reduced, and the higher the chlorophyll content in fresh tea leaves, the more capable they are of forming the quality characteristics of red tea soup and red leaves. Metabolic profiles undergo extensive transformation, and cultivar characteristics influence the efficiency and direction of these transformations. Yellow tea tree varieties can also be made into high-quality black tea. These findings provide new insights and supporting data for the precise utilization of the two tea cultivars and the quality control of black tea.

## 1. Introduction

Ningzhou Gongfu Black Tea, abbreviated as Ninghong Tea, is produced in three counties in Jiangxi Province: Xiushui, Wuning, and Tonggu. It is the earliest Gongfu black tea in China and is renowned for its reputation of “the tea that covers China, the price that ranks first in the world” and “the tea that will not be opened until it reaches the estate” [1,2]. As one of the famous ”Four Greens and One Red” teas in Jiangxi, Ninghong tea is known for its quality characteristics of a tightly twisted shape with prominent golden tips, a dark and lustrous color, a bright red infusion, a long-lasting sweet aroma with a mellow taste, and a bright red leaf base. The tea varieties “Huangjinju” and “Ningzhou No. 2” both originate from Xiushui County in Jiangxi Province. They are systematically bred new clonal varieties [3,4]. “Ningzhou No. 2” has been identified by the National Tea Tree Variety Appraisal Committee and is listed as one of the national superior tea varieties, with green-colored leaves, making it a leading variety of Ninghong. “Huangjinju” is a photosensitive yellowing tea tree resource with yellow young shoots and leaves, named for its chrysanthemum-like appearance [5]. The two varieties have a large planting area in the Xiushui production region and play important roles in tea production and processing in Xiushui County and in the development of the Jiangxi tea industry, especially in Ninghong tea production [6].

Leaf color is a plant phenotypic trait, reflecting the content and relative proportions of natural pigments such as chlorophyll, carotenoids, and anthocyanins [7]. Chlorophyll and carotenoids are lipid-soluble pigments that determine leaf color and tea quality [8]. Numerous studies have demonstrated that chlorophyll synthesis is impaired in leaf color mutants of tea plants [9,10,11,12,13,14]. Research results on carotenoid content in tea plant varieties with color variations differ from those on chlorophyll content. Some studies suggest that color variation leads to increased carotenoid synthesis [15], while others indicate no significant change in total carotenoid content, although carotenoid composition may change, affecting leaf color [16]. Beyond pigments, leaf color mutants often show distinct biochemical components, typically characterized by a higher amino acid content, lower chlorophyll and tea polyphenol contents, and lower phenol-to-amino acid ratios [17]. The variation also extends to aroma components; for instance, “Huangyu” (the yellow mutant of “Yinghong No. 9”) contains fewer free aroma compounds and lower levels of most glycoside-bound aroma precursors compared to normal green leaves [18]. During the fermentation of “Huangyu”, the degradation of carotenoids produces α-farnesene, β-citral, nerol, and trans-β-ionone, which are related to its floral and fruity aroma [19].

Due to their unique phenotypic and biochemical characteristics, tea plants with color variations have broad application prospects in tea variety breeding and tea processing [20]. Strengthening basic research on production and processing applications based on their physiological and biochemical differences is necessary. In this study, “Huangjinju” and its original variety “Ningzhou No. 2” were selected as experimental materials. We determined their main biochemical components and analyzed the key metabolites in fresh leaves and processed black tea using widely targeted metabolomics. This study aims to explore the accumulation differences between the two cultivars, providing a theoretical basis for the processing quality research of yellow-leaf tea plant varieties and a scientific basis for the application of specific tea plant varieties.

## 2. Results

### 2.1. Phenotypic Traits and Pigment Content of HP and NP, and Sensory Characteristics of HT and NT

**Figure 1:**
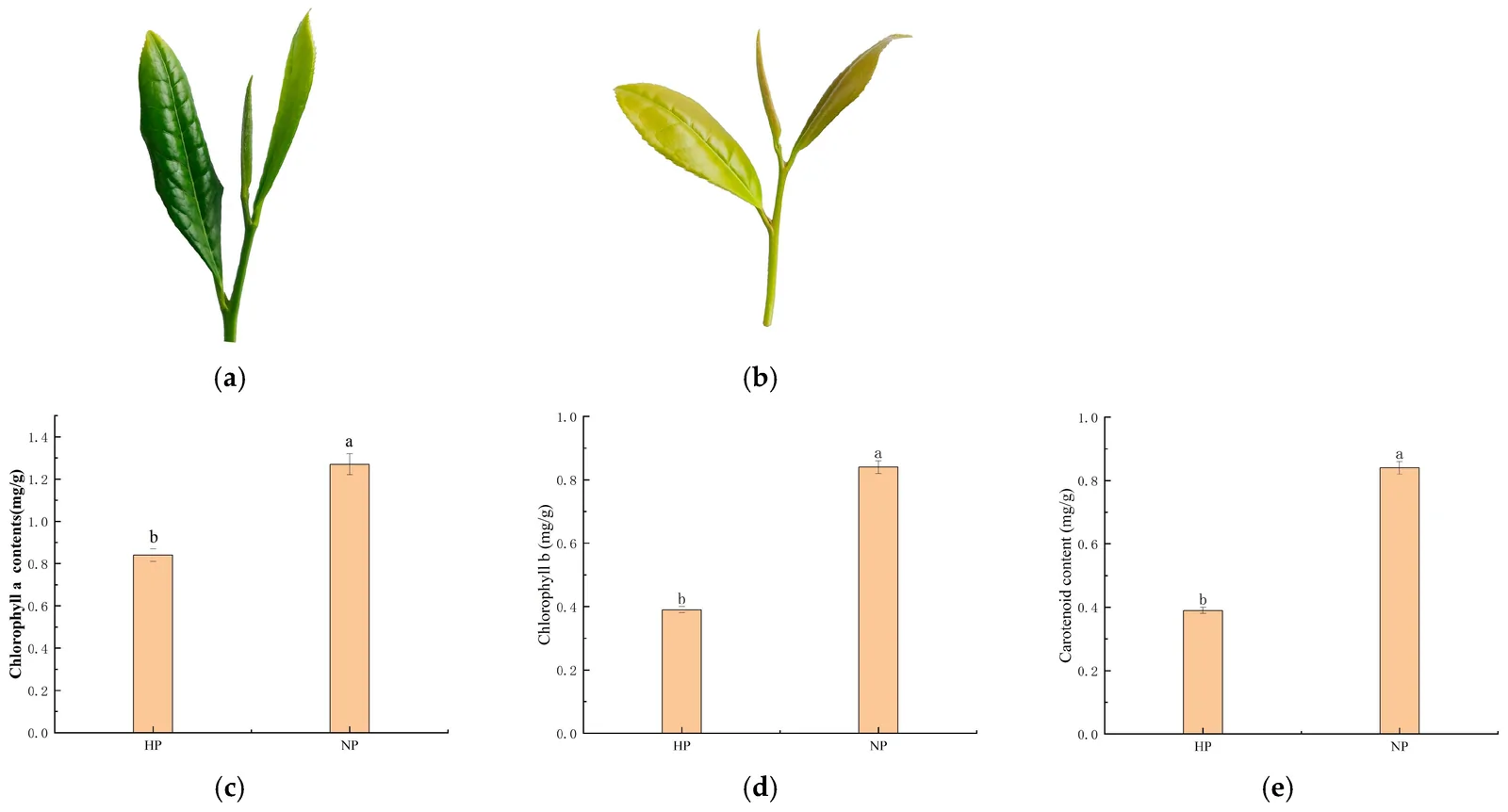
Phenotypes and pigment contents in “Huangjinju” and “Ningzhou No. 2” leaves. (**a**) Leaf phenotypes of “Huangjinju”; (**b**) leaf phenotypes of “Ningzhou No. 2”; (**c**) chlorophyll a content of “Huangjinju” and “Ningzhou No. 2”; (**d**) chlorophyll b content of “Huangjinju” and “Ningzhou No. 2”; (**e**) carotenoid content of “Huangjinju” and “Ningzhou No. 2”. The data are presented as the mean ± standard deviation (n = 3). Means with different letters at each treatment represent a significant difference at *p* ≤ 0.05.

Both “Huangjinju” and “Ningzhou No. 2” fresh leaves were medium-sized and elliptical, but there was a significant difference in leaf color. The color of “Ningzhou No. 2” was green, whereas “Huangjinju” showed distinct yellow leaves (Figure 1a,b). As “Huangjinju” belongs to light-sensitive yellowing tea plant resources, its yellowing phenomenon may be related to a reduction in the number of chloroplasts, or a loosening or decrease in the stacking of thylakoid granules under light stress conditions [14]. Pigment analysis showed that the chlorophyll content in both fresh leaves and processed black tea was significantly lower in “Huangjinju” compared to “Ningzhou No. 2” (Figure 1c,d). The carotenoid content in fresh leaves of “Huangjinju” was also significantly lower than that in “Ningzhou No. 2”, although the difference in carotenoid content in the processed black tea samples between the two varieties was minimal (Figure 1e). Changes in the content of metabolites involved in carotenoid and flavonoid pathways also play an important role in leaf color formation [21].

**Figure 2:**
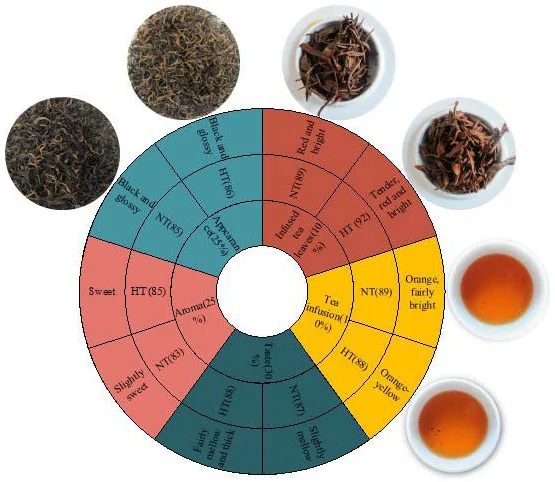
Sensory characteristics in HT and NT (infusion color, infused leaves, appearance, taste, and aroma).

The sensory evaluation results (Figure 2) showed that the traditional black tea NT had an orange-red liquor color, a slightly sweet aroma, a slightly astringent taste, a tight appearance with visible trichomes, and a dark, lustrous color. The HT group was significantly superior to NT in terms of liquor color, sweet and mellow taste, and spent leaves, but received a lower score for tea infusion, presenting an orange-yellow color, and its spent leaves score was reduced to 88 points. The total sensory score for the HT tea sample was 87.15, 1.25 points higher than that for the NT tea sample.

Ninghong tea, produced from fresh tea (*Camellia sinensis*) leaves, is an important, widely popular non-alcoholic beverage due to its health benefits and pleasant flavor. Unlike most other agricultural products, the color of infused leaves is involved in the sensory quality formation of tea. Thus, researchers have recently paid more attention to studying the relationship between leaf color and characteristic compounds, and its influence on health benefits. In fact, compared to typical green leaves, several albino tea varieties have been successfully developed into new products because of their precious and tasteful quality, e.g., *Camellia sinensis* cv. “Anji baicha”, “Yujingxiang”, and “Huangjinya”. Unfortunately, tea breeding goals have been limited to a brighter color or better taste. Other valuable traits, such as yield, flavor value, and manufacturing suitability, have long been ignored [19].

Previous studies indicated that the formation of tea leaf color from white to green might be due to changes in different pigments and their ratios. In general, the greening of leaves was mainly due to higher concentrations of chlorophylls, and the albino-induced white and yellow leaves were mainly due to lower concentrations of chlorophyll, while certain concentrations of carotenoids dominated in leaf formation [6].

The sensory evaluation results also indicate that the yellow bud leaves of HT exhibit a superior tea appearance, liquor color, taste, and aroma. During the processing of black tea, chlorophyll content is significantly reduced, and the higher the chlorophyll content in fresh tea leaves, the more capable they are of forming the quality characteristics of red tea soup and red leaves. Chlorophyll and anthocyanins in fresh leaves can undergo oxidation reactions with polyphenolic substances during the processing of black tea, forming polyphenolic oxides. Carotenoids can also react with other substances during the processing of black tea, thereby promoting the formation of black tea aroma. Therefore, fresh tea leaves with a high pigment content may have a material basis for reacting with polyphenolic substances, which can provide the necessary foundation for the processing of high-quality black tea.

### 2.2. Overview of Metabolites for “Huangjinju” and “Ningzhou No. 2” Samples

**Figure 3:**
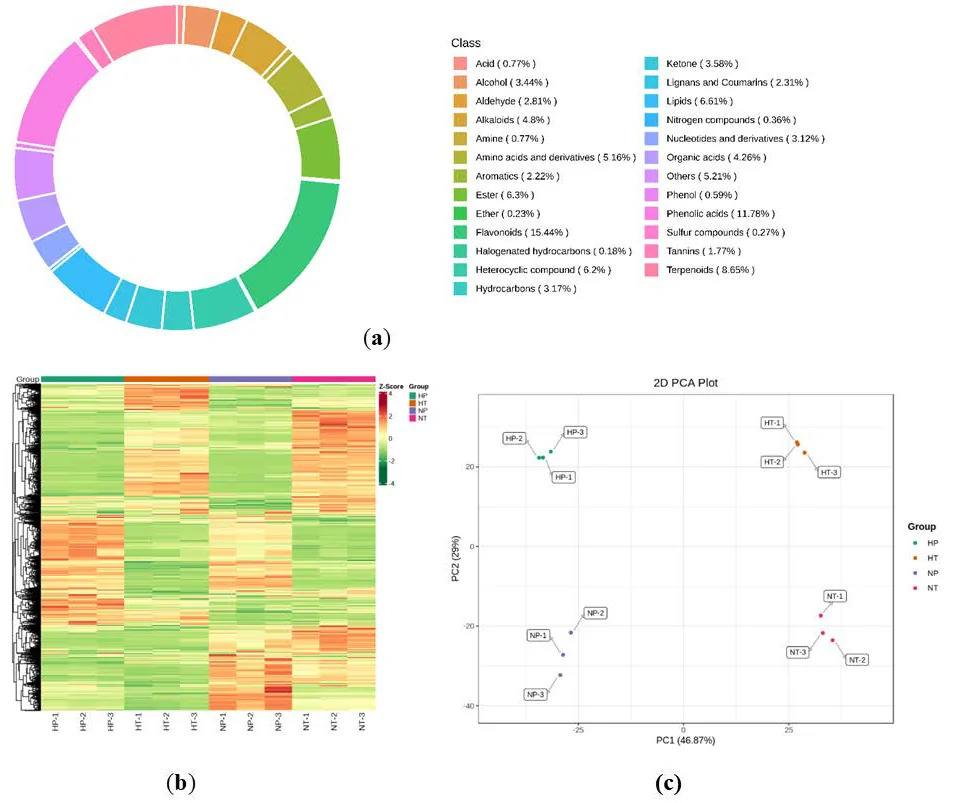
Classification of the 2208 metabolites of tea samples (**a**). Hierarchical cluster analysis (HCA) (**b**) and principal component analysis (PCA) (**c**).

A total of 2208 metabolites were identified in the fresh leaves and processed black tea of “Huangjinju” and “Ningzhou No. 2”, including 872 volatile metabolites and 1336 non-volatile metabolites. They were divided into 25 classes, including acids (0.77%), alcohols (3.44%), aldehydes (2.81%), alkaloids (4.80%), amines (0.77%), amino acids and derivatives (5.16%), aromatics (2.22%), esters (6.30%), ethers (0.23%), flavonoids (15.44%), halogenated hydrocarbons (0.18%), heterocyclic compounds (6.20%), hydrocarbons (3.17%), ketones (3.58%), lignans and coumarins (2.31%), lipids (6.61%), nitrogen compounds (0.36%), nucleotides and derivatives (3.12%), organic acids (4.26%), others (5.21%), phenols (0.59%), phenolic acids (11.78%), sulfur compounds (0.27%), tannins (1.77%), and terpenoids (8.65%) (Figure 3a).

Hierarchical clustering analysis of the metabolites in the tea samples is presented as a heatmap (Figure 3b). The heatmap demonstrated that the three biological replicates of each group clustered tightly into independent branches, confirming excellent sample reproducibility and reliable experimental data.

Principal component analysis (PCA) reduces high-dimensional original data to several principal components to intuitively characterize dataset features and evaluate intra- and inter-group sample differences. In this study, PCA based on the four sample groups revealed clear separation among all treatment groups, indicating substantial intrinsic metabolic differences across samples (Figure 3c). The first principal component (PC1) accounted for 46.87% of the total variance and primarily distinguished samples at different processing stages. The second principal component (PC2) explained 29.00% of the total variance and effectively separated samples of the two tea cultivars.

#### 2.2.1. Non-Volatile Metabolites in Fresh Leaves and Processed Black Tea Samples of “Huangjinju” and “Ningzhou No. 2”

**Figure 4:**
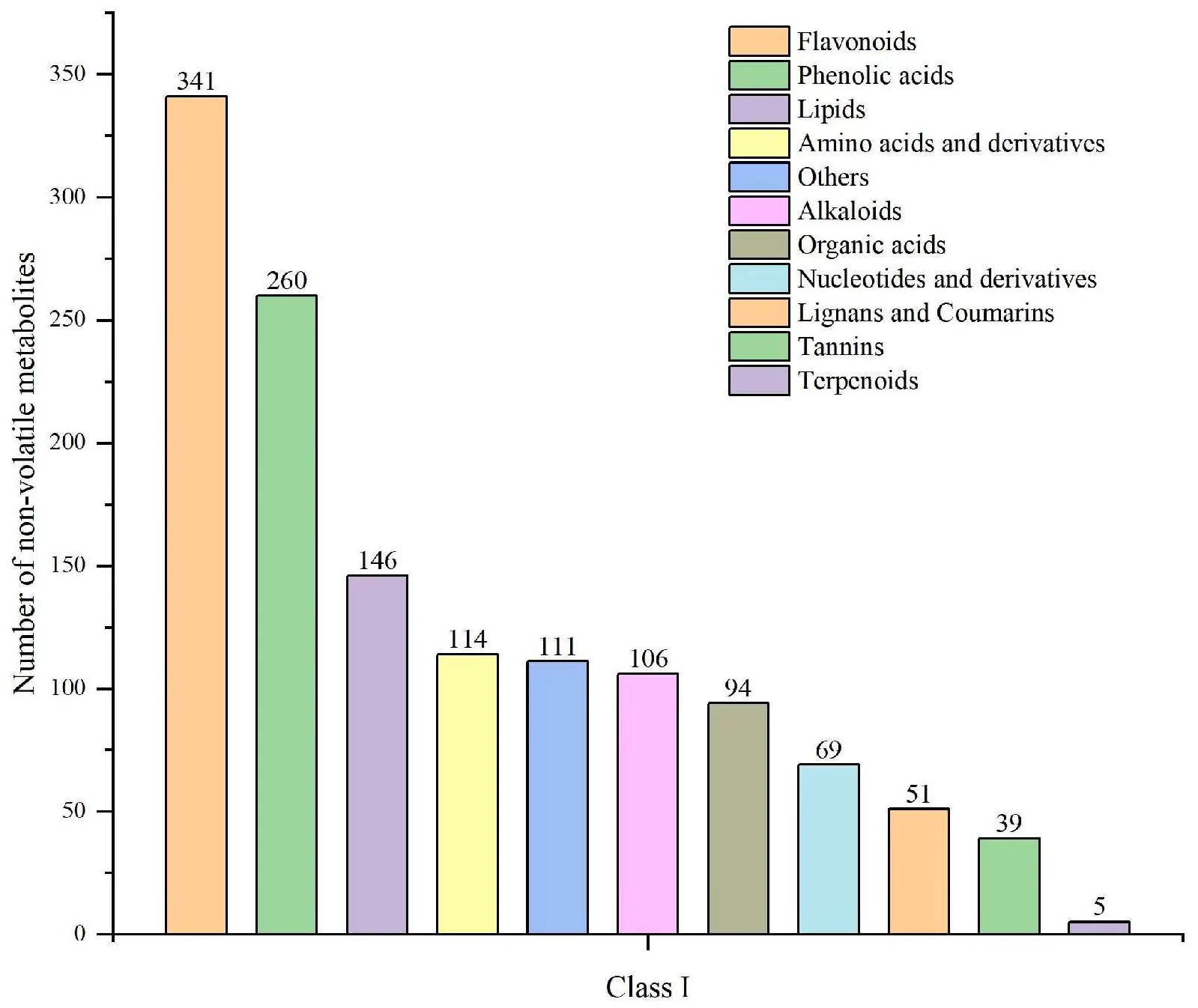
Classification of the non-volatile metabolites of “Huangjinju” and “Ningzhou No. 2”.

A total of 1336 non-volatile metabolites derived from “Huangjinju” and “Ningzhou No. 2” were profiled using the UPLC–MS/MS platform. They encompassed 146 lipids, 94 organic acids, five terpenoids, 106 alkaloids, 39 tannins, 51 lignans and coumarins, 341 flavonoids, 69 nucleotides and their derivatives, 260 phenolic acids, 114 amino acids and their derivatives, and 111 other metabolites (Figure 4).

Flavonoids and flavonoid glycosides are core functional compounds in tea, accounting for 2–3% of the dry weight of tea leaves and exhibiting excellent antioxidant capacity [22]. In this study, three differentially accumulated non-volatile flavonoid compounds with differential contents were screened in the fresh leaves of the two tea cultivars. Specifically, hesperetin-7-O-glucoside, isoquercitrin-7-O-glucoside, and nepetin-7-O-alloside were the predominant flavonoids in fresh “Huangjinju” leaves, whereas quercetin-4’-O-glucuronide, amoenin, and apigenin-7-O-rutinoside exhibited the highest relative abundances in fresh “Ningzhou No. 2” leaves. After black tea processing, nepetin-7-O-alloside remained the dominant flavonoid in processed “Huangjinju” black tea, while quercetin-4’-O-glucuronide maintained the highest level in processed “Ningzhou No. 2” black tea (Table S1).

Phenolic acids are aromatic carboxylic compounds with multiple phenolic hydroxyl groups substituted on a benzene ring and serve as crucial contributors to tea flavor formation [23]. They contribute to the acidic taste of tea soup and are major bioactive components with various biological activities, including strong antioxidant, anti-tumor, and antibacterial properties. Compared to catechins and flavonoids, phenolic acids are more readily absorbed by the body [24]. In this study, the differential phenolic acid components in fresh leaves between the two varieties were identified as digalloylglucose and 2-O-galloyl-D-glucose, with a higher relative content in the fresh leaves of “Huangjinju”. The differential phenolic acid components in the processed black teas of the two varieties included cimidahurinine,5-(2-Hydroxyethyl)-2-O-glucosylphenol, 1,6-Di-O-caffeoyl-4-O-galloyl-β-D-glucose, caftaric acid, and 1,3,4,6-Tetra-O-galloyl-β-D-glucose. After processing into black tea, the phenolic acid content in “Ningzhou No. 2” increased (Table S1).

**Figure 5:**
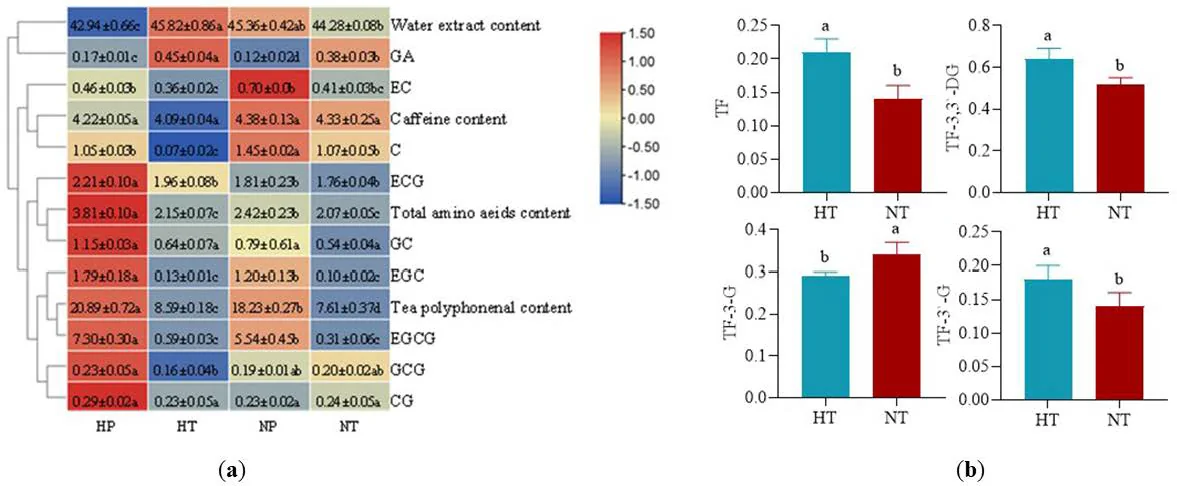
Cluster analysis of the biochemical contents heatmap in “Huangjinju” and “Ningzhou No. 2”. Data are presented as mean ± standard deviation. Different lowercase letters indicate significant differences among groups (*p* < 0.05). (**a**) comparative analysis of flavor components, (**b**) comparison of monomeric components of tea pigments.

Biochemical component analysis showed that the content of tea polyphenols (tea polyphenol content), catechins (such as ECG, EGCG, etc.), and total amino acids (total amino acid content) in fresh leaves (HP and NP) were significantly higher than those in processed black tea (HT and NT). The components, such as water extract content and caffeine content, in processed black tea were relatively more prominent. At the same time, there were also differences among varieties. For example, the contents of catechins and amino acids in the fresh leaves of “Huangjinju” (HP) were higher than those in the fresh leaves of “Ningzhou No. 2” (NP), reflecting the dual effects of processing fresh leaves into black tea and the characteristics of varieties on the content of components. Processing reduced most active components, while varieties determined the basic content differences of components (Figure 5a).

The content and proportion of characteristic theaflavins in processed black tea showed significant differences due to different varieties. The content of TF, TF-3,3’-DG, and TF-3’-G in “Huangjinju” black tea (HT) was significantly higher than that in “Ningzhou No. 2” (NT), while the content of TF-3-G in “Ningzhou No. 2” black tea (NT) was significantly higher than that in “Huangjinju” black tea (HT) (Figure 5b). The biochemical components of tea plants form the material basis for tea quality, and variations in leaf color in tea plants could be associated with significant differences in biochemical component levels compared to conventional varieties. In recent years, tea plant mutants with specific leaf colors have attracted increasing attention due to their unique appearance and quality [16]. The amino acid content in yellowing varieties is significantly higher than in the control varieties [25], with tea amino acids constituting the highest proportion of amino acids [26]. Compared to green-leaf varieties, these yellowing varieties have better economic value [27]. Research results showed that the chlorophyll and carotenoid contents in fresh leaves of “Huangjinju” were significantly lower than those in “Ningzhou No. 2”. However, the tea polyphenol and amino acid contents in “Huangjinju” were significantly higher than those in “Ningzhou No. 2”. “Huangjinju” belongs to light-sensitive, yellowing tea plant resources. Abnormal chloroplast development or loss may disrupt the carbon–nitrogen metabolism balance in the plant, ultimately leading to an increase in free amino acid content and a decrease in polyphenol content [28].

Amino acids are organic compounds containing amino and carboxyl groups in tea [29]. They impart a certain freshness and aroma to tea, making the tea soup more refreshing and mellow [30,31]. The free amino acid content in the fresh leaves of “Huangjinju” was significantly higher than in the fresh leaves of “Ningzhou No. 2”. However, after processing into black tea, there was no significant difference in amino acid content between the two varieties. Among the detected differential metabolites, trimethyllysine had a higher relative content in the fresh leaves of “Huangjinju”. After processing into black tea, the relative content of S-methyl glutathione significantly decreased in both varieties, while the relative content of L-glutamine-O-glucoside increased in “Ningzhou No. 2” (Table S2).

#### 2.2.2. Volatile Metabolites in Fresh Leaves and Processed Black Tea Samples of “Huangjinju” and “Ningzhou No. 2”

**Figure 6:**
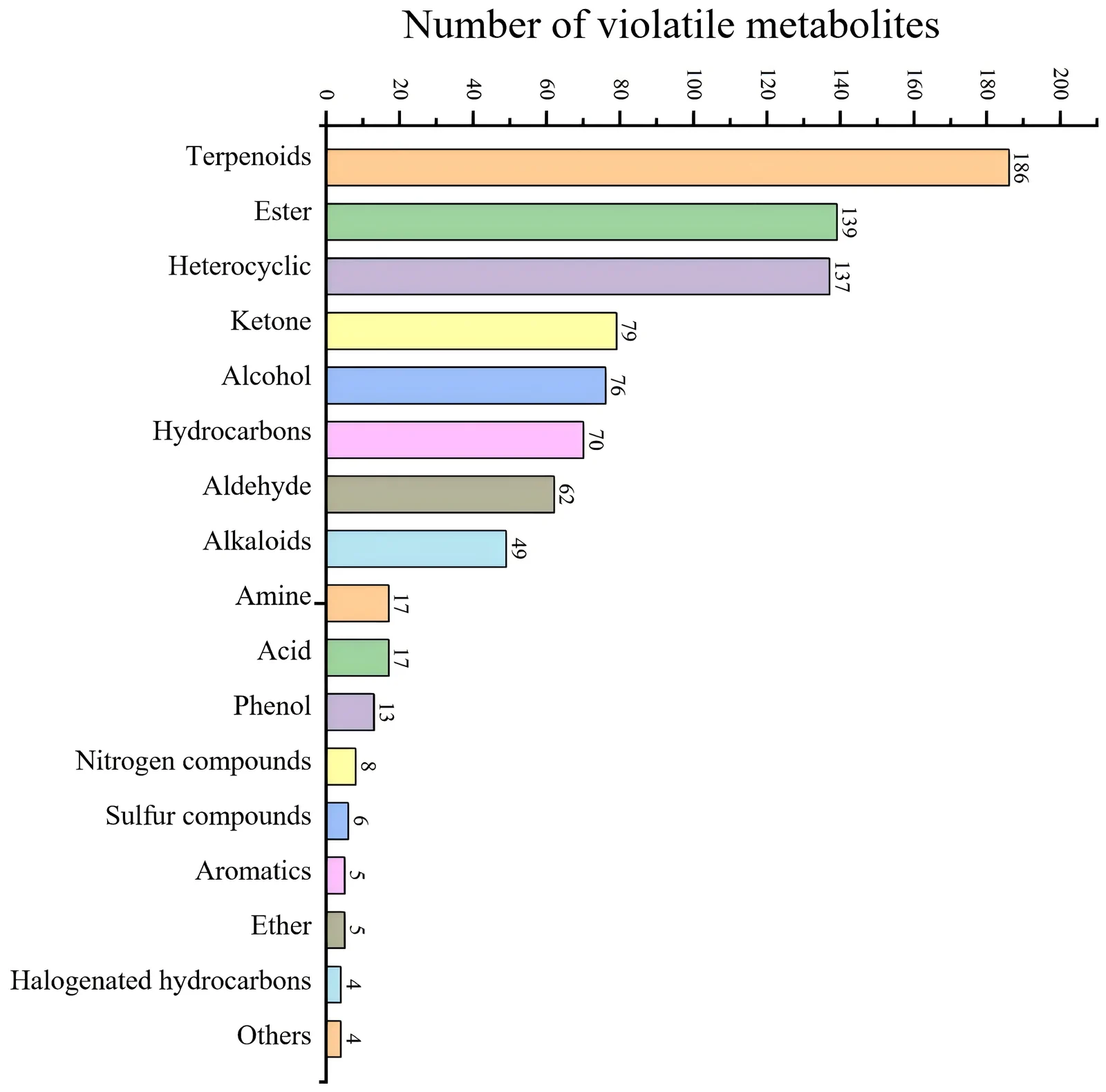
Classification of the volatile metabolites of “Huangjinju” and “Ningzhou No. 2”.

A total of 872 volatile metabolites detected from “Huangjinju” and “Ningzhou No. 2” were analyzed via the HS–SPME–GC–MS platform by Wuhan Maivei Metabolic Biotechnology Co., Ltd. The identified metabolites were classified into 17 categories, including 17 amines, 76 alcohols, four aromatics, 49 alkaloids, 113 phenolics, eight nitrogen-containing compounds, six sulfur-containing compounds, four halogenated hydrocarbons, five ethers, four others, 62 aldehydes, seven acids, 186 terpenoids, 70 hydrocarbons, 79 ketones, 137 heterocyclic compounds, and 139 esters (Figure 6).

The flavor contribution of volatile compounds to the characteristic aroma of black tea depends not only on their concentrations in the tea infusion but also on their odor activity values (OAVs). The OAV is defined as the ratio of a compound’s concentration to its odor threshold, which quantifies the individual contribution of each volatile compound to the overall tea flavor profile. Compounds with OAV ≥ 1 are generally considered key aroma-active substances that effectively shape sensory perception [32]. In this study, the OAVs of all detected volatiles were calculated based on previously reported aqueous odor thresholds (Table 1), and metabolites with OAV ≥ 1 were screened as potential aroma-active compounds in processed black tea. Comparative analysis revealed that multiple aroma-related volatiles, including 3,7-dimethyl-(Z)-1,3,6-octatriene, α-phellandrene, 1-methyl-4-(1-methylethyl)-1,1,3-cyclohexadiene, trans-β-ocimene, β-ionone, (E,E)-2,4-heptadien-1-ol, 2-furanmethanol, 3-mercapto-3-methylbutanol, and 1-octen-3-ol, exhibited higher relative abundances in “Ningzhou No. 2” than in “Huangjinju”, with several of these compounds exceeding their aqueous odor thresholds (OAV > 1). In contrast, processed black tea manufactured from “Huangjinju” contained higher levels of trans,cis-2,6-nonadien-1-ol, (2S,4R)-4-methyl-2-(2-methylprop-1-en-1-yl)tetrahydro-2H-pyran, and N,N-dimethylbenzenamine, which are primarily responsible for its unique aroma characteristics.

### 2.3. Screening of Differential Metabolites

**Figure 7:**
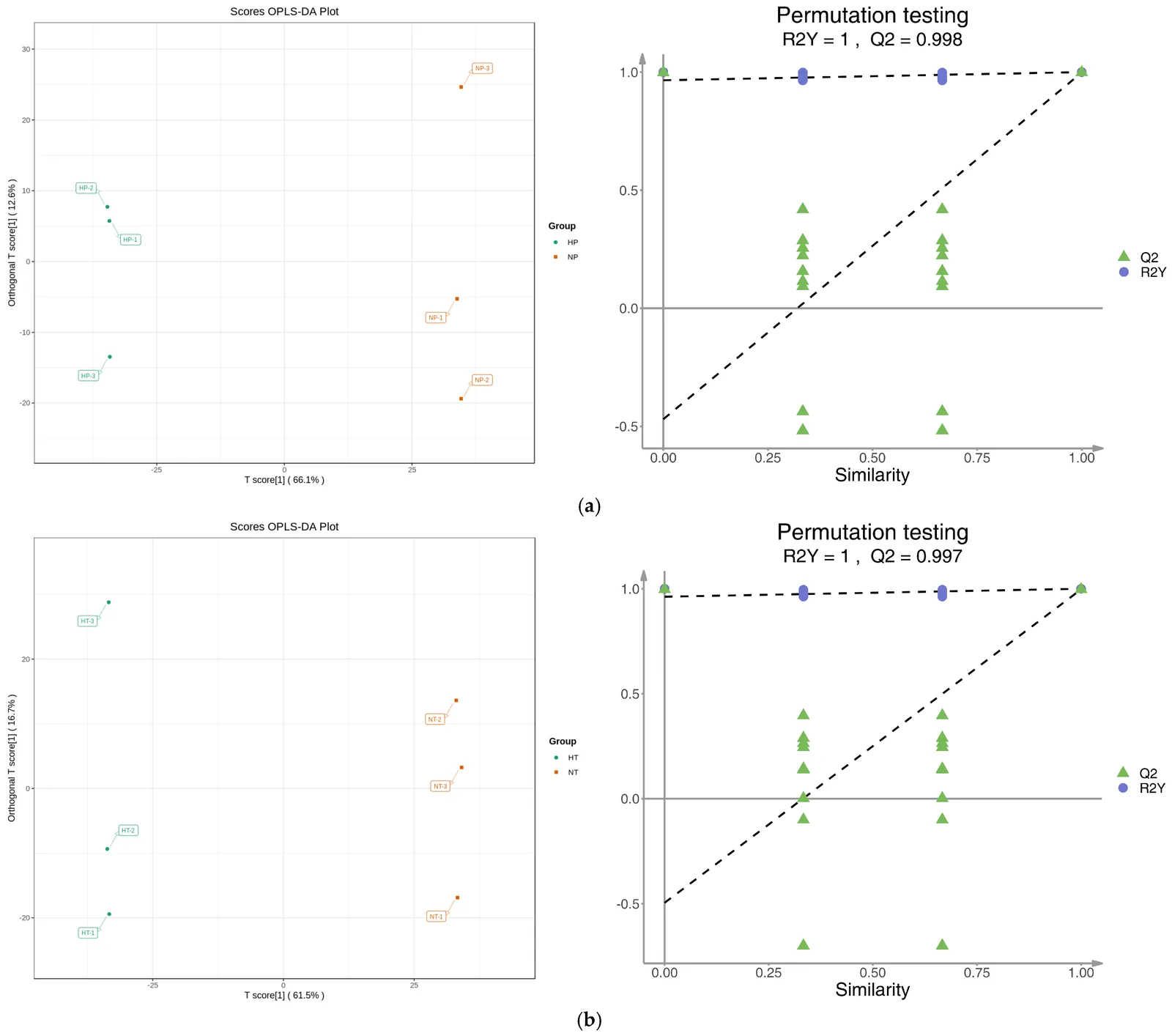
The score plots of OPLS-DA pairwise comparisons of differential metabolites. (**a**) HP vs. NP; (**b**) HT vs. NT.

Orthogonal partial least-squares discriminant analysis (OPLS-DA) is a supervised multivariate statistical approach that can eliminate irrelevant variational noise, thereby maximizing group separation and enabling the reliable screening of differential metabolites. Pairwise comparisons of HP vs. NP and HT vs. NT were achieved using the OPLS-DA model, and the score plots are presented in Figure 7. The results revealed obvious metabolic discrimination between groups for the two tea cultivars, with all comparison groups achieving significant separation.

**Figure 8:**
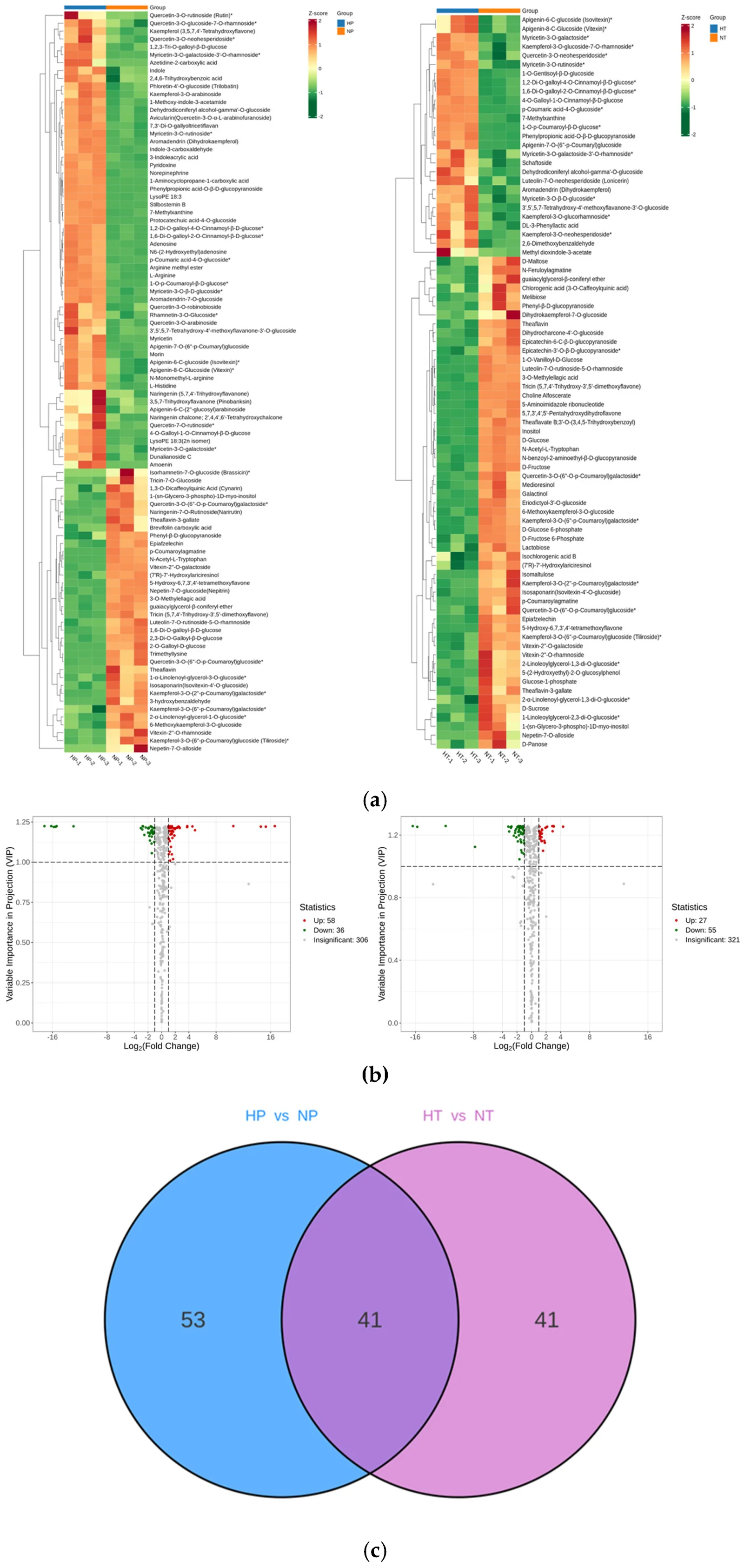
(**a**) The heat map of comparisons (HP vs. NP and HT vs. NT); (**b**) The volcano plot of differential metabolites from comparisons (HP vs. NP and HT vs. NT); (**c**) The Venn diagram of comparisons (HP vs. NP and HT vs. NT). Asterisks (*) indicate metabolites with significant differences between groups (*p* < 0.05).

Differential metabolites were preliminarily screened according to the thresholds of fold change (FC ≥ 2 or FC ≤ 0.5) and variable importance in projection (VIP ≥ 1). It should be noted that these screening thresholds were applied without supplementary *p*-value or false discovery rate (FDR) correction; thus, the obtained metabolites represent only putative metabolic variations rather than statistically significant differences. A Venn diagram was generated to visualize the overlapping and specific differential metabolites between comparison groups. In total, 41 common differential metabolites were identified in fresh leaves of “Huangjinju” and “Ningzhou No. 2”, representing core cultivar-specific metabolic markers. Furthermore, 53 metabolites differed exclusively between the two cultivars at the fresh leaf stage (HP vs. NP), while 41 metabolites showed cultivar-specific differences only after black tea processing (HT vs. NT), indicating that black tea processing substantially reshapes metabolic divergence between the two tea cultivars. Heatmap and volcano plot analyses further intuitively illustrated the distinct accumulation patterns of these putative differential metabolites in the fresh leaves and processed black tea of the two cultivars (Figure 8).

**Figure 9:**
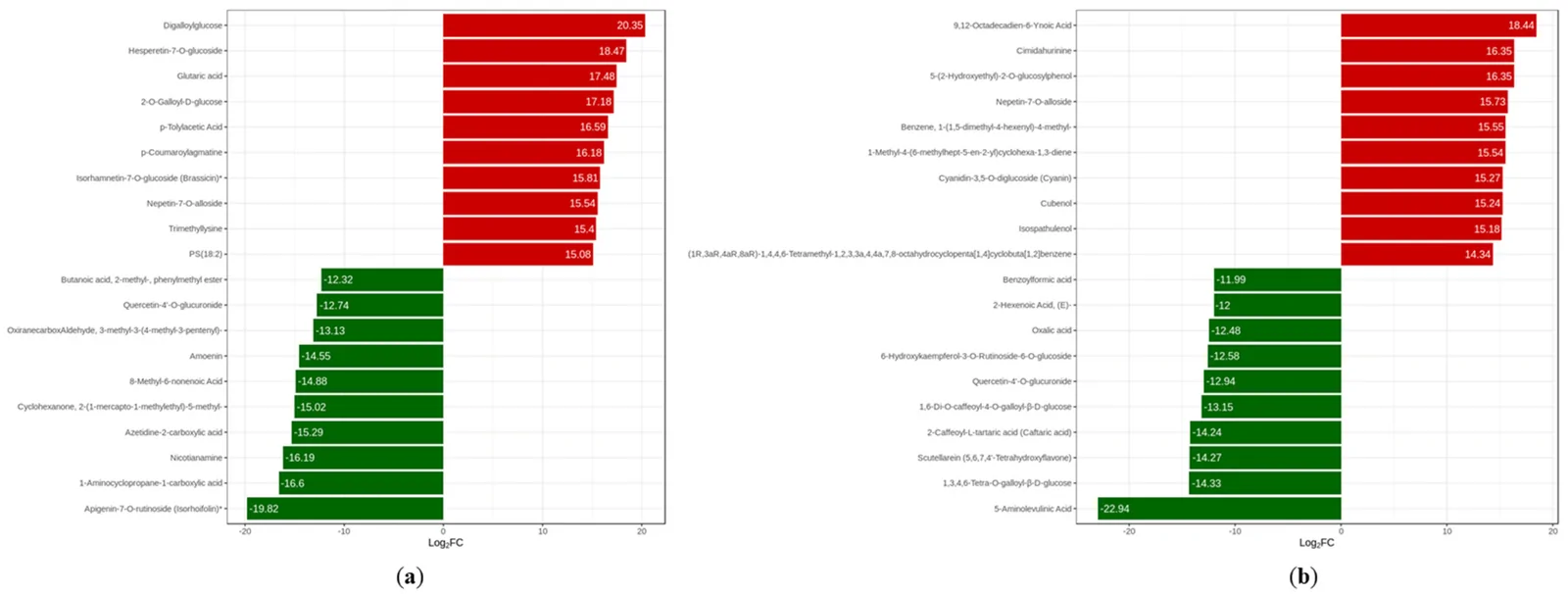
The top FC distribution compounds of comparisons. (**a**) HP vs. NP; (**b**) HT vs. NT.

To further elucidate the metabolic variations between the two tea cultivars and across different processing stages, FC values of all annotated metabolites were calculated, and the top 10 significantly upregulated and downregulated metabolites in each comparison group were summarized. Flavonoids were identified as the predominant differential metabolites distinguishing fresh leaves of the two cultivars. Most metabolites that were downregulated in fresh “Huangjinju” leaves relative to “Ningzhou No. 2” presented extremely low abundances or were even undetectable in “Ningzhou No. 2”, indicating distinct cultivar-specific metabolic accumulation patterns. In processed black tea, 5-aminolevulinic acid exhibited the most significant downregulation between the two cultivars. Overall, terpenoid metabolites were highly accumulated in processed black tea of “Huangjinju”, whereas flavonoids and phenolic acids dominated the differential metabolic profiles of “Ningzhou No. 2” processed black tea (Figure 9).

Monoterpenes (C10) and sesquiterpenes (C15) serve as core aroma-active compounds contributing to tea fragrance, while flavonoids and phenolic acids are primary polyphenolic substances that contribute to the mellow taste of the tea infusion [33,34,35]. Accordingly, the distinct accumulation of terpenoids, flavonoids, and phenolic acids is presumed to be the primary metabolic foundation underlying the divergent aroma and taste characteristics of black tea manufactured from the two cultivars.

**Figure 10:**
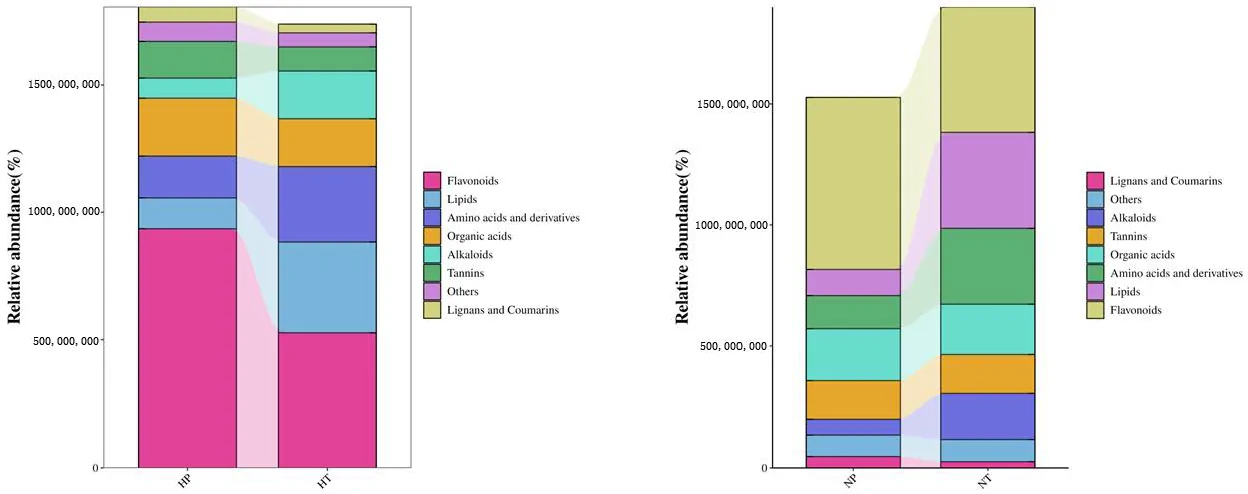
The metabolite category compositions and subclass trend chart of comparisons.

Dynamic metabolomic variations of the two cultivars throughout black tea processing were comprehensively analyzed based on metabolite compositional profiles and subclass accumulation trends (Figure 10). Substantial shifts in the relative proportions of major metabolite classes were observed after processing. For “Huangjinju”, processing induced a reduction in the relative abundance of flavonoids and an elevation in lipid proportions. In contrast, processed “Ningzhou No. 2” black tea exhibited increased flavonoid abundance, accompanied by significant enrichment of lipids, amino acids, and their derivatives. These results demonstrate cultivar-specific metabolic responses to processing-induced stress: processed “Huangjinju” black tea is characterized by prominent lipid accumulation, whereas processed “Ningzhou No. 2” black tea displays dominant flavonoid enrichment.

All annotated metabolites were clustered into four subclasses with distinct accumulation patterns. Subclass 1 (134 metabolites) was highly accumulated in processed tea samples (HT and NT), whereas Subclass 3 (97 metabolites) showed high accumulation in fresh leaf samples (HP and NP), indicating unique metabolite accumulation profiles that are collectively modulated by cultivar genotype and processing treatment.

**Figure 11:**
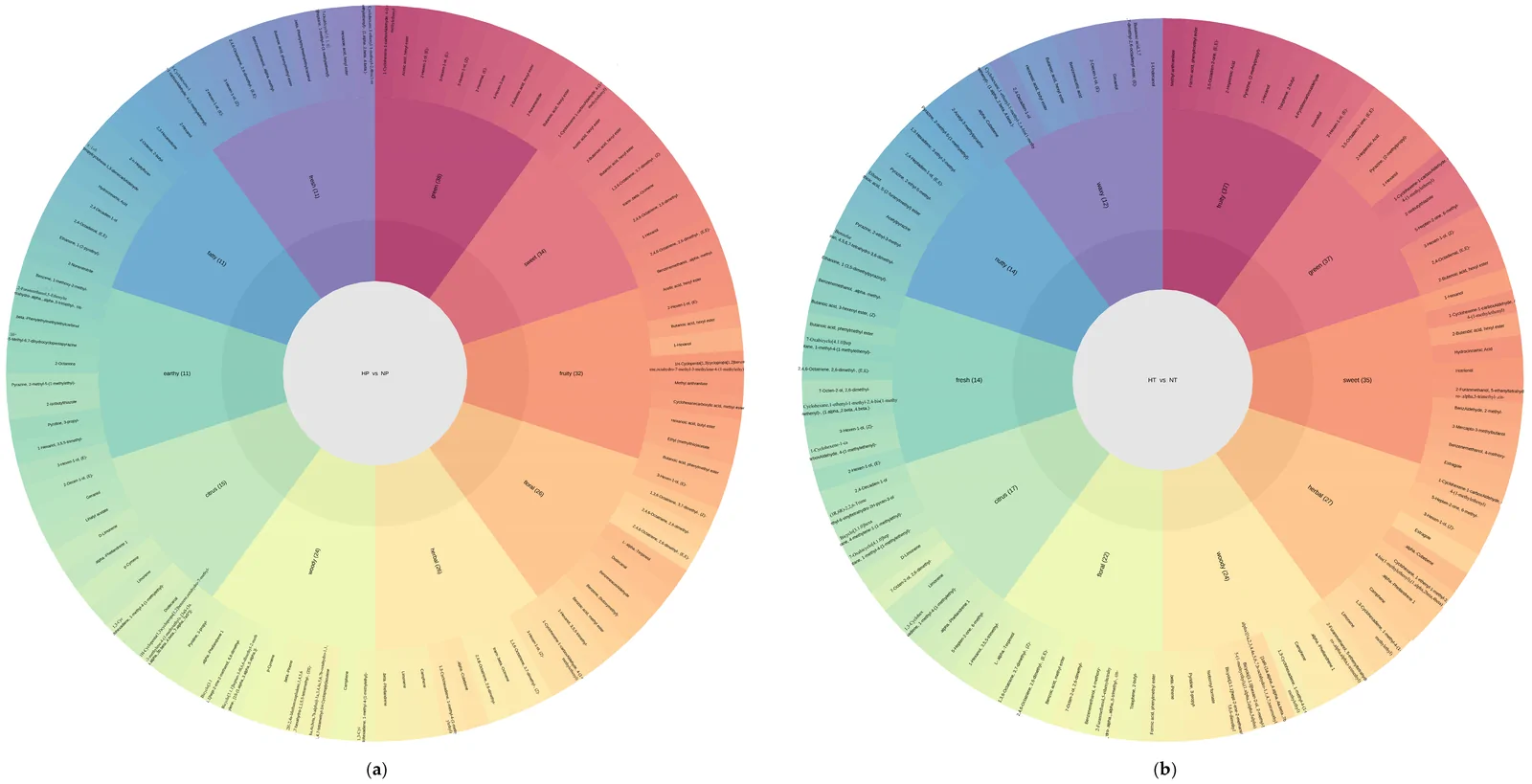
Flavor wheel of differential metabolites. (**a**) Flavor wheel of differential metabolites between HP and NP; (**b**) flavor wheel of differential metabolites between HT and NT. Note: The innermost ring indicates the differential comparison group. The second ring shows the top 10 sensory flavor characteristics (ranked by the highest number of annotated differential metabolites) to which the differential metabolites in that comparison group were mapped; the numbers in parentheses indicate the number of differential metabolites mapped to each sensory flavor characteristic. The outermost ring represents the differential metabolites. When the number of differential metabolites mapped to a given sensory flavor characteristic exceeds 10, only the top 10 differential metabolites with the highest VIP values are displayed.

Based on the preliminarily screened differential metabolites and their annotated flavor features, a total of 324 flavor-active metabolites were identified and retrieved (Table S3). The top 10 flavor categories with the most annotated metabolites were visualized via a flavor wheel (Figure 11a,b). The flavor profiles of the four groups covered green, fruity, sweet, woody, floral, herbal, and citrus notes. Among these, green, sweet, and fruity flavors accounted for more than 50% of all flavor-related metabolites, constituting the dominant aroma characteristics of fresh tea leaves. In the processed tea comparison, the HP vs. HT group exhibited enriched floral and nutty flavor-related metabolites but contained fewer spicy and apple aroma components relative to the NP vs. NT group.

Notably, six volatile metabolites with OAV ≥ 1 were screened as potential characteristic flavor compounds in the HT vs. NT group (corrected from the original typo “HY vs. NT”), including cubenol, isospathulenol, (1R,3aR,4aR,8aR)-1,4,4,6-tetramethyl-1,2,3,3a,4,4a,7,8-octahydrocyclopenta [1,4]cyclobuta [1,2]benzene, 1-(1,5-dimethyl-4-hexenyl)-4-methylbenzene, and 1-methyl-4-(6-methylhept-5-en-2-yl)cyclohexa-1,3-diene, which were significantly upregulated, whereas (E)-2-hexenoic acid was downregulated. As a typical naturally occurring sesquiterpene alcohol widely detected in aromatic plants, cubenol endows tea with prominent floral and rose-like fragrances. The high accumulation of this sesquiterpene compound contributes substantially to the intense floral, rosy, and sweet aroma characteristics of “Huangjinju” black tea.

### 2.4. KEGG Annotation and Enrichment Analysis of Differential Metabolites

**Figure 12:**
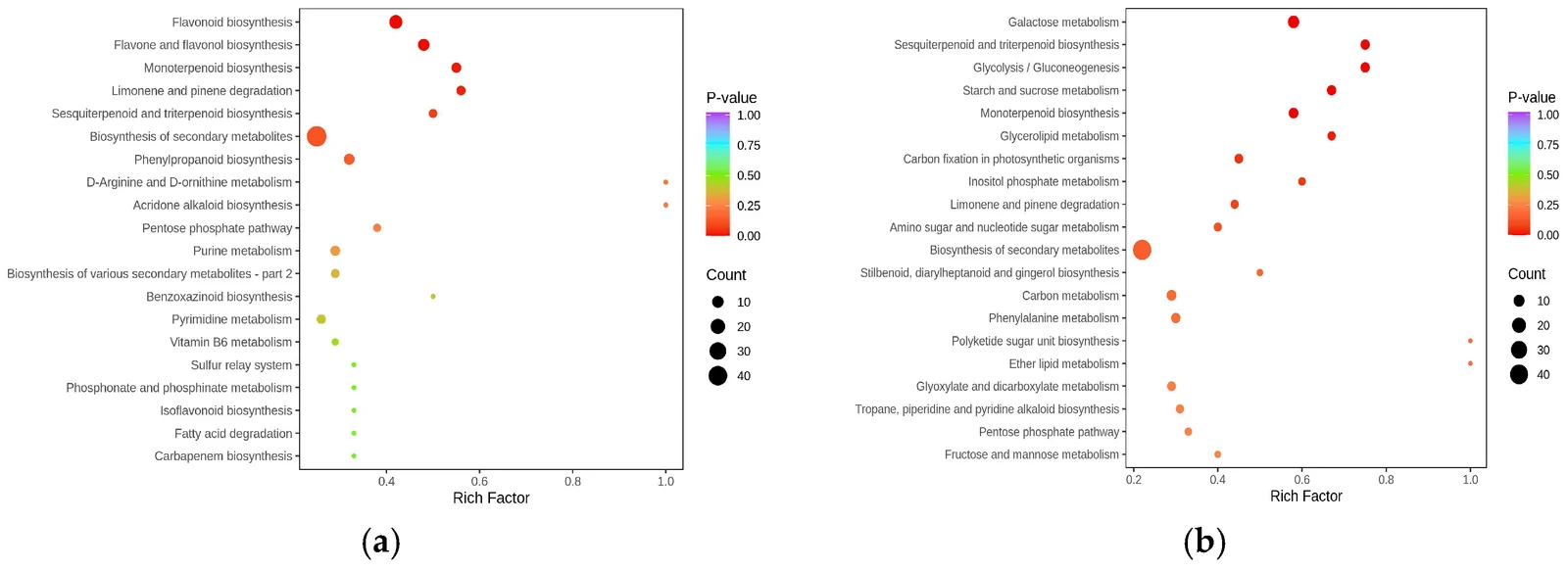
KEGG annotations and enrichment of differentially expressed metabolites of each pairwise comparison of tea samples. (**a**) HP vs. NP; (**b**) HT vs. NT.

KEGG annotation and enrichment analysis were performed on differential metabolites screened from each pairwise comparison of tea samples (Figure 12a,b). Pathway enrichment analysis demonstrated that the differential metabolites derived from fresh leaves of the two tea cultivars were predominantly enriched in secondary metabolism pathways, mainly flavonoid biosynthesis and terpenoid biosynthesis. Among these, flavonoid biosynthesis and flavone and flavonol biosynthesis were the most significantly enriched pathways.

In the flavonoid biosynthesis pathway, hesperetin 7-O-glucoside and (−)-epiafzelechin were upregulated, whereas 13 metabolites were downregulated (Figure S1a). In the flavone and flavonol biosynthesis pathway, vitexin 2″-O-beta-L-rhamnoside and quercitrin were upregulated, with eight metabolites showing decreased accumulation (Figure S1b). Metabolite profiling analysis confirmed distinct cultivar-specific flavonoid accumulation signatures. Fresh leaves of “Huangjinju” exhibited prominent enrichment of flavonol metabolites, including kaempferol and quercetin, whereas fresh leaves of “Ningzhou No. 2” accumulated higher levels of isoflavone and anthocyanin metabolites. These divergent metabolite accumulation characteristics indicated differential sub-pathway preferences of flavonoid metabolism between the two tea cultivars.

In processed black tea, cultivar-specific differential metabolites were predominantly enriched in multiple key metabolic pathways, including galactose metabolism, sesquiterpenoid and triterpenoid biosynthesis, glycolysis/gluconeogenesis, starch and sucrose metabolism, and monoterpenoid biosynthesis. The differential enrichment of these pathways provides a potential metabolic foundation for cultivar-dependent variations in carbohydrate turnover and terpenoid anabolism and catabolism, which further shape the divergent taste and aroma quality of finished black tea products. Galactose metabolism was identified as the core differential pathway, with 12 metabolites exhibiting significantly increased accumulation between the two cultivars (Figure S1c).

The two black tea cultivars presented distinct metabolite accumulation patterns at key nodes of the galactose metabolism pathway, indicating inherent differences in downstream biochemical regulation. Notably, the present study only detected steady-state metabolite abundances, without quantitative validation of enzyme activities or gene expression levels. Processed “Huangjinju” black tea exhibited higher accumulation of galactitol and lactose, suggesting an intrinsic metabolic tendency toward galactitol biosynthesis. In contrast, “Ningzhou No. 2” black tea tended to convert galactose into glycolytic precursors such as glucose. Collectively, the divergent accumulation of sugar-related metabolites reflects cultivar-specific regulatory disparities in sugar metabolism, which ultimately differentiates the metabolic profiles and comprehensive quality characteristics of the two black tea cultivars.

## 3. Discussion

This study provides comprehensive metabolic insight into the quality differentiation mechanism between the yellow-leaf cultivar “Huangjinju” and the conventional green-leaf cultivar “Ningzhou No. 2”. The present findings demonstrate that the distinct yellow phenotype of “Huangjinju” may not merely represent a superficial visual trait but could be intrinsically associated with profound biochemical reprogramming. This phenotypic variation is characterized by suppressed biosynthesis of chlorophylls and carotenoids, as well as the selective accumulation of critical quality-related metabolites (e.g., free amino acids and tea polyphenols) in fresh tea leaves [36].

Such metabolic and phenotypic differences between the two cultivars become more prominent after processing via the standard Ningzhou Gongfu black tea manufacturing protocol. The conventional Ninghong processing procedure yields finished black tea with tightly rolled leaves, a glossy yellow–green leaf appearance, and reddish stems. The standardized processing parameters adopted in this study included prolonged withering (18 h at 26–30 °C with 55–65% relative humidity), an additional turning-over cycle, graded rolling for 1.0–2.0 h at 32 rpm following the “light–heavy–light” principle, fermentation (3–4 h at 25–28 °C with 95–96% relative humidity), and multi-stage drying (primary and final drying) until the leaf moisture content decreased below 6%. Collectively, intensive moisture loss and elevated oxidation during standardized Ninghong processing substantially promote tea liquor color formation and pigment accumulation. Tea plant genotype serves as a decisive factor shaping the comprehensive quality attributes of finished black tea. Specifically, the cultivar’s genetic background modulates the intensity of the bitterness, aroma complexity, and sweet taste perception of the tea infusion, acting as a core limiting factor for black tea quality formation [37,38]. Notably, the “Huangjinju” cultivar endows processed black tea with a superior morphological appearance, aromatic profiles, and taste characteristics, showing great potential for producing high-quality Ningzhou Gongfu black tea.

Widely targeted metabolomics analysis was applied to characterize the metabolic divergence between the two cultivars, tentatively identifying a total of 2208 metabolites classified into 25 categories. Multivariate statistical analyses, including PCA and OPLS-DA, were able to effectively discriminate tea samples based on cultivar variation and processing treatment. Black tea processing may act as a critical regulatory factor in metabolic modulation, potentially amplifying certain inherent cultivar-specific metabolic differences while attenuating others [39,40]. For example, the divergent accumulation patterns of major metabolite classes (e.g., flavonoids tended to decrease in processed “Huangjinju” black tea but increase in processed “Ningzhou No. 2” black tea) indicate distinct genotype-dependent metabolic responses to processing-induced stress.

Metabolic pathway enrichment analysis further revealed the intrinsic mechanisms underlying these metabolic differences. Fresh “Huangjinju” leaves exhibited a preferential metabolic flux toward the flavonol branch of the flavonoid biosynthesis pathway [41,42]. In processed black tea, differential metabolites between the two cultivars were predominantly enriched in galactose metabolism and terpenoid biosynthesis pathways. These pathway-specific metabolic alterations are functionally essential, as they reshape the accumulation of key precursor substances governing tea taste and aroma during processing. Specifically, carbohydrate metabolism regulates the sweet sensory attribute of the tea infusion, while terpenoid biosynthesis determines the formation of core aromatic compounds. Based on odor activity value (OAV) quantification of volatile components, a set of cultivar-specific key aroma-active compounds was screened, which collectively define the unique sensory characteristics of the two tea cultivars.

The MEP (methylerythritol 4-phosphate) pathway, related to the synthesis of terpenoid aromas such as monoterpenes and sesquiterpenes, also needs to be implemented in chloroplasts and other plastids. The abnormal chloroplasts in “Huangjinju” inevitably affect normal leaf photosynthesis and metabolite accumulation, and also impact the synthesis, accumulation, and transformation of aroma-related metabolites to some extent. We found that the percentages of aroma substances in “Ningzhou No. 2”, which included 1,3,6-octatriene,3,7-dimethyl-,(Z), alpha-phellandrene, 1,1,3-cyclohexadiene, 1-methyl-4- (1-methylethyl), trans-beta.-ocimene, β-ionone, 2,4-heptadien-1-ol,(E,E), 2- furanmethanol, 3-mercapto-3-methylbutanol, and 1-octen-3-ol, were significantly higher than those in “Huangjinju” and exceeded their detection thresholds in water. The contents of aroma substances in “Huangjinju” processed black tea were much greater than those in “Ningzhou No. 2”, including trans,cis-2,6-nonadien-1-ol, (2S,4R)-4-methyl-2-(2-methylprop-1-en-1-yl)tetrahydro-2H-pyran, benzenamine, and N,N-dimethyl, which are the key aroma substances in “Huangjinju”.

In conclusion, this study may provide a metabolic reference for the efficient utilization of specific tea plant cultivars and could offer theoretical guidance for the product development and industrial production of Jiangxi Congfu black tea.

## 4. Materials and Methods

### 4.1. Plant Materials and Sample Preparation

This study was conducted during the spring of 2023. Fresh tea leaves (one bud and two leaves) were harvested from 23-year-old *Camellia sinensis* cv. “Huangjinju” and “Ningzhou No. 2”. The samples were collected from the ecological tourism tea garden base of the Jiangxi Economic Crops Research Institute in Nanchang City, China (116.01° E, 28.37° N).

**Figure 13:**
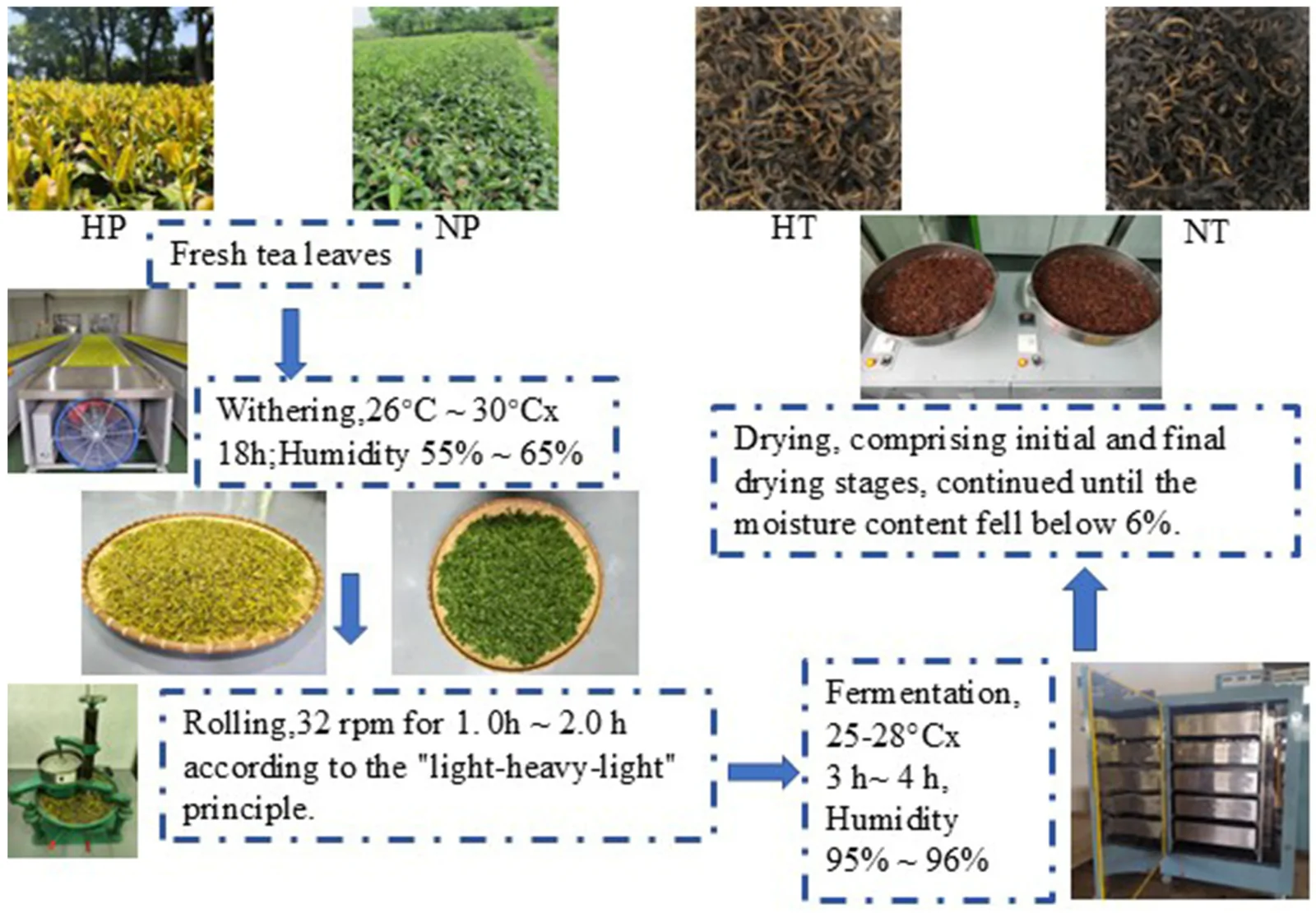
The schematic diagram of black tea processing. Note: “Huangjinju” fresh leaves (HP), “Ningzhou No. 2” fresh leaves (NP), “Huangjinju” processed black tea (HT), and “Ningzhou No. 2” processed black tea (NT).

Black tea processing followed DB36/T 1794-2023, the <Technical Specification for Processing of Congou Black Tea>. Fresh leaves were subjected to indoor natural withering for 18 h until they became soft and lost their luster, with a water content of 55–65%. The rolling process was performed following the “light–heavy–light” principle by a rolling machine for 60–120 min. The fermentation was conducted using a fermentation machine for 3–4 h at a temperature of 25–28 °C, with a relative humidity of over 90%, until the leaves turned yellow-red and developed a fresh floral and fruity aroma. After fermentation, the tea leaves were dried using a 6CTH-8.0 dryer machine (Zhejiang Zhu Feng Machinery Co., Ltd., Quzhou, China) at an initial drying time of 10 min at 110 °C, a redrying time of 30 min at 90 °C, and a final drying at 80 °C for 60 min, with sufficient cooling to room temperature and rewetting between each drying step. The drying process consisted of initial and final drying until the water content was reduced to below 6%. The dried black tea sample must weigh more than 200 g. The dried tea was stored at −20 °C in a freezer until further analysis (Figure 13).

### 4.2. Sampling

The simple random sampling method was used to collect samples from both the fresh leaves and processed black tea of the two varieties. The samples included “Huangjinju” fresh leaves (HP), “Ningzhou No. 2” fresh leaves (NP), “Huangjinju” processed black tea (HT), and “Ningzhou No. 2” processed black tea (NT). All samples, including finished black teas (after final sorting), were prepared in triplicate (approximately 250 g per replicate) for subsequent analyses. To ensure authenticity and reproducibility, the entire manufacturing process was supervised by a professional tea master with over 10 years of experience, who adjusted processing parameters in real time according to leaf morphology and aroma development. Fresh leaves (HP and NP) were immediately frozen with liquid nitrogen and stored in a freezer at −80 °C for the following analysis.

### 4.3. Pigment Content Determination

The determination of chlorophyll and carotenoid contents referred to the method in reference [4]. Briefly, 100 randomly harvested fresh young shoots from “Huangjinju” fresh leaves (HP) and “Ningzhou No. 2” fresh leaves (NP) were ground, and 0.2 g of the ground product was extracted with 10 mL acetone and 0.1 g polyvinylpolypyrrolidone. The mixture was centrifuged at 12,000 rpm for 15 min (4 °C). The supernatant was analyzed on the LC-20AT HPLC System (Shimadzu, Kyoto, Japan) with a TC-C18 column (Agilent Technologies Inc., Santa Clara, CA, USA). The injection volume was 20 μL. The column was eluted at 35 °C with a linear gradient increasing from 80 to 100% mobile phase B (acetonitrile/methanol/chloroform:15/4/1, ***v***/*v*/*v*) over 20 min at a flow rate of 1 mL min^−1^. After an additional 15 min at 100% mobile phase B, the gradient was linearly decreased from 100 to 80% over 5 min, and then 80% mobile phase B for an additional 5 min. The absorbance at wavelengths of 663 nm, 645 nm, and 470 nm was measured; the blank control used was 95% ethanol. The calculation formulas for the content of chlorophyll a, chlorophyll b, and carotenoids were as follows:

(1) $$chl\;a=\frac{(12.7\times D_{663}-2.69\times D_{645})\times 10}{M}$$

(2) $$chl\;b=\frac{(22.9\times D_{645}-4.68\times D_{663})\times 10}{M}$$

(3) $$Car=\frac{\frac{\left(10,000\times D_{470}\right)}{M}-3.27\times Chla-104\times Chlb}{198}$$

where D663, D645, and D470 represent the absorbance at 663, 645, and 470 nm; M represents the weight of the samples (g). The contents of chlorophyll and carotenoids were estimated in mg/g.

### 4.4. Main Biochemical Component Measurement

Free amino acid content was determined according to the national standard (GB/T 8314–2013). Briefly, fresh shoots (at the stage with one bud and two leaves) were fixed by steaming for three minutes. Fixed leaves were then dried at 80 °C for 3 h. Dried leaves were ground into a powder and passed through a 0.45mm mesh sieve. Then, 3.0 g of the fine powder was placed in 450 mL of boiling water for 45 min to make the extract solution. The extract solution was then filtered through Double-Ring No. 102 filter paper (Xinhua Paper Industry Co. Ltd., Hangzhou, China), and the volume was increased to 500 mL by adding distilled water. Next, 1 mL of the solution was transferred to a 25 mL flask, followed by the addition of 0.5 ml of buffer (pH 8.0) containing 63 mM Na_2_HPO_4_ and 3 mM KH_2_PO_4_, and 0.5 ml of a 2% ninhydrin solution (2 g ninhydrin and 80 mg SnCl_2_·2H_2_O dissolved in 100 mL of water). The flask was incubated at boiling temperature for 15 min. The volume was then increased to 25 mL with H_2_O. The absorbance (570 nm) of the mixture was measured with a UV Spectrophotometer U-2800 (Hitachi High-Technologies Corporation, Tokyo, Japan). Total free amino acid content was calculated from a standard curve generated with varying concentrations of glutamine. The tea polyphenols, caffeine, and catechin contents were determined according to GB/T 8313-2018 <Determination of total polyphenol and catechin contents in tea>. The water extract content was measured according to GB/T 8305-2013 <Tea—Determination of water extract content>.

### 4.5. Sensory Evaluation

Sensory evaluation was performed in accordance with GB/T 23376–2018 and GB/T 14487–2017, which are the Chinese national standards for tea sensory methodology and vocabulary. Congou black tea samples were homogenized and randomized before assessment. Coded dry-leaf samples were evaluated for visual characteristics. For each evaluation, 3 g of tea was brewed with 150 mL of boiling water in standard cupping vessels, covered, and steeped for 5 min. The infusion was transferred to white porcelain bowls, and assessments of aroma and taste were conducted at 30 s intervals. A panel of five trained tasters independently scored each sample using a 100-point scale: 25% for dry-leaf appearance, 10% for liquor color, 25% for aroma, 30% for taste, and 10% for infused-leaf quality. To ensure consistency, five certified assessors verified the sensory attributes. Afterwards, the appearance, aroma, liquor color, taste, and brewed leaves were evaluated by five experienced assessors authenticated by professional organizations.

### 4.6. Analysis of Non-Volatile Metabolites in Black Teas Using UPLCMS/MS

#### 4.6.1. Extraction of Non-Volatiles

The tea samples were vacuum freeze-dried and then ground into powder. A mass of 50 mg of each sample was weighed, and 70% aqueous methanol extraction solvent containing the internal standard, pre-cooled to −20 °C, was added. This was followed by vortex mixing for 30 s, which was repeated six times with a 30 min interval between each mixing. The mixture was then centrifuged (at 12,000 rpm for 3 min), and the supernatant was aspirated. The sample was filtered using a microporous membrane (with a pore size of 0.22 μm), and the filtered sample was stored in the sample vial for UPLC–MS/MS analysis.

#### 4.6.2. LC–MS Conditions

LC conditions: chromatographic column: Agilent SB-C18 1. 8 µm, 2. 1 mm × 100 mm; Mobile phase: Phase A is ultra-pure water (with 0. 1% formic acid added), Phase B is acetonitrile (with 0.1% formic acid added); elution gradient: 5% B proportion at 0.00 min, linearly increases to 95% within 9.00 min, remains at 95% for 1 min, drops to 5% from 10.00 to 11.10 min, and reaches 5% again for 14 min; flow rate: 0.35 mL/min; column temperature: 40 °C; and injection volume: 4 μL.

MS conditions: electrospray ionization (ESI) temperature: 550 °C; ion spray voltage (IS): 5500 V (positive-ion mode)/−4500 V (negative-ion mode); ion source gas I (GSI), gas II (GSII), and curtain gas (CUR) are set to 50, 60, and 25 psi, respectively, and the collision-induced ionization parameters are set to high. QQQ scanning uses the MRM mode, and the collision gas (nitrogen) is set to medium. Through further optimization of declustering potential (DP) and collision energy (CE), the DP and CE of each MRM ion pair were determined. Based on the metabolites eluted in each period, a specific set of MRM ion pairs was monitored in each period.

#### 4.6.3. Qualitative and Quantitative Analysis of Metabolites Using LC–MS

Based on the self-built database MWDB (metware database), metabolites were qualitatively identified using secondary spectral information. During the analysis, isotope signals were removed, and duplicate signals of K^+^, Na^+^, and NH^4+^ ions, as well as duplicate signals of fragment ions of substances that are themselves other larger-molecular-weight substances, were also eliminated. Specific methods followed the procedure referenced in [21].

### 4.7. Analysis of Volatile Metabolites in Black Teas Using GC–MS

#### 4.7.1. Extraction of Volatiles

The tea samples were ground into powder using liquid nitrogen, and 500 mg of each was placed in a 20 mL headspace bottle. The bottle contained a saturated sodium chloride solution and 20 μL of 3-hexanone-2,2,4,4-d4 (as an internal standard solution) at a concentration of 10 μg/mL.

#### 4.7.2. HS–SPME Extraction Conditions

Each sample vial was agitated for 5 min at 60 °C. A 120 µm DVB/CWR/PDMS extraction head was inserted into the headspace bottle of the sample. The headspace extraction was conducted for 15 min. The sample was then analyzed at 250 °C for 5 min, followed by GC–MS separation and identification. The extraction head was aged at 250 °C in the Fiber Conditioning Station for 5 min before sampling.

#### 4.7.3. GC–MS Conditions

GC conditions: DB-5MS capillary column (30 m × 0.25 mm × 0.25 μm, Agilent J&W Scientific, Folsom, CA, USA), the carrier gas is high-purity helium (purity not less than 99.999%), constant flow rate is 1.2 mL/min, injection port temperature is 250 °C, non-split injection, solvent delay of 3.5 min. Temperature program: 40 °C for 3.5 min, then increased to 100 °C at 10 °C/min, to 180 °C at 7 °C/min, and finally to 280 °C at 25 °C/min, and maintained for 5 min.

MS conditions: electron impact ion source (EI), ion source temperature: 230 °C, quadrupole temperature: 150 °C, mass spectrometry interface temperature: 280 °C, electron energy: 70 eV, scanning mode is the selected ion detection mode (SIM), qualitative and quantitative ion precise scanning (GB 23200.8-2016).

#### 4.7.4. Metabolite Identification and Quantification Principles of GC–MS

Based on multiple species, the literature, partial standards, and retention indices, an independent database was established. The specific qualitative method referred to in [42] was used. Quantitative ions were selected for the integration and correction of chromatographic peaks to enhance the accuracy of quantification.

### 4.8. OAV Calculation

The OAV of each volatile metabolite is calculated by dividing the concentration by the odor threshold. The odor threshold data are derived from previous studies [43] and the database (https://www.odour.org.uk), while the odor descriptions are referenced from the database (https://www.thegoodscentscompany.com/).

### 4.9. Statistical Analysis

All chemical tests were repeated three times, and the LC–MS and HS–SPME–GC–MS analyses of each sample were conducted in three parallel determinations. The results were expressed as the mean ± standard deviation. Significance analysis was performed using SPSS 27, with a *p*-value < 0.05 set as the significance level. The heatmap was generated using TBtools-II software version 2.376. Multivariate statistical analysis of metabolomics data (UPLC-MS/MS and GC-MS) was processed through the MetWare Cloud platform (https://cloud.metware.cn/).

## Figures and Tables

**Table 1:** Volatile compounds and OAVs of “Huangjinju” and “Ningzhou No. 2”.

Compounds	Class I	CAS	Odor Threshold	HP	HT	NP	NT
**1**,3,6-Octatriene, 3,7-dimethyl-, (Z)-	Terpenoids	3338-55-4	0.055	1.372 ± 0.138	0.988 ± 0.226	4.736 ± 0.662	3.557 ± 0.095
alpha.-Phellandrene	Terpenoids	99-83-2	0.16	0.100 ± 0.012	0.069 ± 0.012	0.275 ± 0.036	0.208 ± 0.004
1,3-Cyclohexadiene, 1-methyl-4-(1-methylethyl)-	Terpenoids	99-86-5	0.08	0.137 ± 0.013	0.093 ± 0.019	0.399 ± 0.043	0.319 ± 0.012
trans-.beta.-Ocimene	Terpenoids	3779-61-1	0.034	2.992 ± 0.303	2.555 ± 0.548	9.942 ± 1.338	7.683 ± 0.146
(2S,4R)-4-Methyl-2-(2-methylprop-1-en-1-yl)tetrahydro-2H-pyran	Terpenoids	3033-23-6	0.0005	147.977 ± 2.698	46.916 ± 5.119	115.182 ± 10.720	44.678 ± 3.698
β-Ionone	Terpenoids	14901-07-6	0.021	1.977 ± 0.347	1.885 ± 0.635	1.866 ± 0.583	2.448 ± 0.385
2,4-Heptadien-1-ol, (E,E)-	Alcohol	33467-79-7	0.032	0.701 ± 0.018	0.039 ± 0.006	1.143 ± 0.051	0.109 ± 0.008
2-Furanmethanol	Alcohol	98-00-0	0.0123	0.430 ± 0.068	0.290 ± 0.014	1.566 ± 0.719	0.299 ± 0.036
2-Octanol	Alcohol	123-96-6	0.0715	0.036 ± 0.001	0.000 ± 0.00	0.053 ± 0.001	0.000 ± 0.000
3-Mercapto-3-methylbutanol	Alcohol	34300-94-2	0.002	0.266 ± 0.033	0.072 ± 0.003	0.615 ± 0.167	0.180 ± 0.017
1-Octen-3-ol	Alcohol	3391-86-4	0.016	0.347 ± 0.025	0.088 ± 0.009	0.473 ± 0.091	0.182 ± 0.016
trans,cis-2,6-Nonadien-1-ol	Alcohol	28069-72-9	0.001	17.225 ± 0.894	12.687 ± 2.573	14.098 ± 3.200	11.629 ± 1.978
1-Nonanol	Alcohol	143-08-8	0.09	0.014 ± 0.001	0.050 ± 0.006	0.016 ± 0.002	0.081 ± 0.006
Benzene, 1,2,4,5-tetramethyl-	Aromatics	95-93-2	0.083	0.212 ± 0.021	0.076 ± 0.003	0.227 ± 0.035	0.095 ± 0.010
2,4-Decadienal, (E,E)-	Aldehyde	25152-84-5	0.027	0.025 ± 0.003	0.021 ± 0.008	0.029 ± 0.008	0.049 ± 0.012
BenzenAmine, N,N-dimethyl-	Amine	121-69-7	0.012	0.905 ± 0.015	0.209 ± 0.018	0.633 ± 0.073	0.193 ± 0.017

## Data Availability

No new data were created or analyzed in this study. Data sharing is not applicable to this article.

## Supplementary Materials

The following supporting information can be downloaded at: https://www.mdpi.com/article/10.3390/ijms27188313/s1.

## Abbreviations

The following abbreviations are used in this manuscript:

OAV	Odor Activity Value
PCA	Principal Component Analysis
OPLS-DA	Orthogonal Partial Least-Squares Discriminant Analysis
VIP	Variable Importance in Projection
GA	Gallate
EC	Epicatechin
C	Catechin
ECG	Epicatechin Gallate
GC	Gallocatechin
EGC	Epigallocatechin
EGCG	Epigallocatechin Gallate
GCG	Gallocatechin gallate
CG	Catechin gallate
